# Topography of Juxtaventricular white matter hyperintensities and cognitive associations in Alzheimer's disease: a dual-cohort study

**DOI:** 10.1186/s41747-026-00776-1

**Published:** 2026-07-29

**Authors:** Yi-Wen Bao, Ya-Qing Ji, Li-Na Wang, Yu Zhou, Fen Wang, Da-Ming Shen, Dmytro Pylypenko, Qiang Tong, Ka-Fung Mak, Li-Li Guo

**Affiliations:** 1 Department of Medical Imaging, The Affiliated Huai’an No.1 People’s Hospital of Nanjing Medical University, Northern Jiangsu Institute of Clinical Medicine, Nanjing Medical University, Huai’an, China; 2 Department of Medical Imaging, The Affiliated Huai’an No.1 People’s Hospital of Nanjing Medical University, Huai’an, China; 3 Huai’an Clinical College of Xuzhou Medical University, Huai’an, China; 4 Department of Neurology, The Affiliated Huai’an No.1 People’s Hospital of Nanjing Medical University, Huai’an, China; 5 GE Healthcare, Shanghai, China; 6 Department of Diagnostic Radiology, Li Ka Shing Faculty of Medicine, The University of Hong Kong, Hong Kong, China

**Keywords:** Alzheimer disease, Cerebral small vessel diseases, Cognitive dysfunction, Magnetic resonance imaging, White matter

## Abstract

**Objective:**

White matter hyperintensities (WMHs), a hallmark of cerebral small vessel disease, frequently coexist with Alzheimer's disease (AD) and facilitate cognitive deterioration. Juxtaventricular WMH (JVWMH) is hypothesized to reflect pathological processes at the cerebrospinal fluid (CSF)-parenchyma interface and hold particular clinical significance. Therefore, this study specifically investigated associations between JVWMH burden and cognitive performance, CSF volume (CSFV), and amyloid-β (Aβ) pathology.

**Materials and methods:**

Automated WMH segmentation was applied in two cohorts: 295 from the Australian Imaging, Biomarkers and Lifestyle (AIBL) study and 82 from a memory clinic. All participants underwent 3-T magnetic resonance imaging, amyloid-positron emission tomography, and cognitive assessment. Analyses evaluated JVWMH associations with Aβ, its discriminative value for cognitive impairment (CI) *versus* cognitively normal (CN), prediction of longitudinal decline, and mediation of CSFV-cognition relationships.

**Results:**

JVWMH volume significantly discriminated CI from CN participants in both cohorts and predicted cognitive decline in longitudinal AIBL data. JVWMH volume showed strong correlations with CSFV in both cohorts. Notably, JVWMH partially mediated the associations between CSFV and cognition in the AIBL cohort. Non-juxtaventricular WMHs and Fazekas scores demonstrated no significant diagnostic or predictive power.

**Conclusion:**

JVWMH volume provides diagnostic and prognostic information beyond conventional WMH metrics in AD. Its correlation with CSFV implicates CSF-parenchyma interface processes, though causality remains unproven. These findings highlight the importance of incorporating spatially stratified WMH metrics in AD research to better capture disease-relevant white matter pathology.

**Key Points:**

- **Question:** JVWMH volume provides diagnostic and prognostic value beyond conventional WMH metrics in AD.
- **Findings:** JVWMH volume discriminated CI from normal cognition, predicted longitudinal decline, correlated with CSF volume, and partially mediated CSF volume–cognition associations.
- **Relevance statement:** JVWMH volume provides diagnostic and prognostic information beyond conventional WMH metrics in AD, highlighting the value of spatially stratified WMH analysis in AD research to better capture disease-relevant white matter pathology.

**Graphical Abstract:**

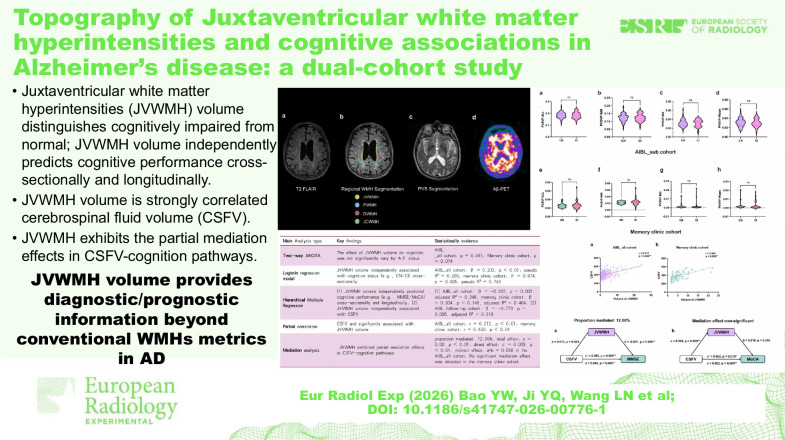

## Background

Alzheimer's disease (AD) is the leading cause of dementia, characterized by progressive cognitive decline and neurodegeneration [1]. Cerebral small vessel disease frequently coexists with AD, contributing to cognitive deterioration and complicating differential diagnosis [2, 3]. White matter hyperintensities (WMHs), a key manifestation of cerebral small vessel disease, are commonly observed on T2-weighted fluid-attenuated inversion recovery (FLAIR) magnetic resonance imaging (MRI) in patients with AD and mild cognitive impairment (MCI) [4].

Increasing evidence links AD neuropathology with vascular abnormalities, demonstrating associations between WMH severity and β-amyloid (Aβ) or tau deposition [5–7]. Higher WMH burden has been associated with cognitive impairment (CI) and increased dementia risk [8]. Histopathological, neuroimaging, and genetic studies collectively indicate a multifactorial etiology for WMHs, with region-specific variation in WMH pathogenesis across brain areas [9, 10].

Conventional classification divides WMHs into periventricular and deep subtypes [11], however, these categories rely on inconsistent operational and anatomical definitions, limiting reproducibility across studies [12, 13]. To address these limitations, a spatially stratified WMH framework has been proposed that partitions lesions into juxtaventricular WMHs (JVWMH), periventricular WMHs (PVWMH), deep WMHs (DWMH), and juxtacortical WMHs (JCWMH) [14, 15], thereby enhancing lesion characterization and clinical relevance. This hierarchical approach reduces heterogeneity within the periventricular and deep WMHs categories and provides improved diagnostic and prognostic value.

WMHs arise from heterogeneous processes. Previous reports suggested that JVWMH, which are located immediately adjacent to the ventricular lining, may arise from disruptions at the cerebrospinal fluid (CSF)-parenchyma interface, such as ependymal damage, altered CSF flow, or ventricular morphology changes, in addition to microvascular injury [1, 2]. Enlarged perivascular spaces (PVS) and altered CSF dynamics [3] have been increasingly documented along the AD continuum [4–6].

Building on these findings, the present study aimed to: (1) demonstrate associations between JVWMH volume and Aβ burden; (2) determine whether JVWMH shows stronger associations with diagnostic status and cognitive performance compared with other WMH subtypes across two independent cohorts; and (3) explore its relationships with CSF volume (CSFV), including exploratory mediation.

## Methods

### Participants

Participants were recruited from two independent cohorts: (1) a university-affiliated memory clinic and (2) the Australian Imaging, Biomarkers and Lifestyle (AIBL) Study of Ageing (AIBL, https://aibl.org.au). This study was approved by the respective ethics committees (memory clinic cohort IRB: UW 11-1260), and all participants provided written informed consent.

The AIBL cohort included 295 participants: 194 cognitively normal (CN), 52 MCI, and 49 AD patients, collectively termed the **AIBL_all** cohort. The **memory clinic** cohort comprised 82 participants: 15 CN, 9 subjective cognitive decline, 33 MCI, and 25 AD patients. A subset of 84 AIBL participants with follow-up data was defined as the **AIBL_follow-up** cohort, where cognitive decline was determined as a 3-or more-point reduction (MMSE) score over time [7]. Among the 84 subjects in follow-up, 42 subjects underwent subsequent MRI scans.

**Fig. 1:**
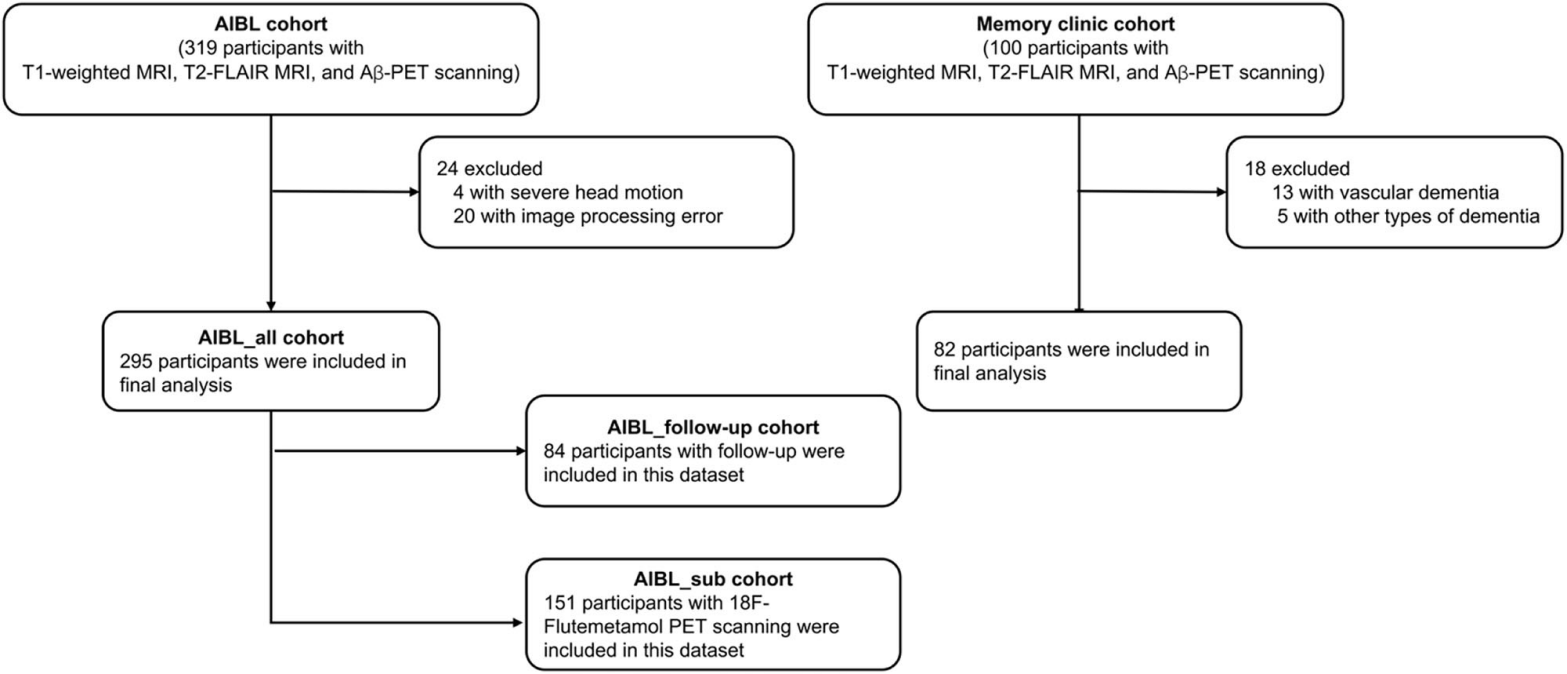
Flowchart visualizing the selection process of participants

One hundred fifty-one AIBL participants who underwent ^18^F-Fluetemtamol Aβ-positron emission tomography (PET) scanning were selected for PVS analyses and defined as the **AIBL_sub** cohort. Participant selection is summarized in Fig. 1. Notably, 20 participants (all from the AIBL cohort) were excluded because the automated image processing pipeline failed at one or more stages for their MRI data, such as segmentation failures or registration failures, rendering them unusable for quantitative analysis.

All participants underwent clinical evaluation, neuropsychological tests, T1-weighted (T1w) MRI, T2-FLAIR MRI, and Aβ-PET scanning. Participants with CN or subjective cognitive decline were combined as the CN group, while those with MCI or AD were classified as the CI group.

### Inclusion criteria

Memory clinic cohort: CN participants were healthy elderly individuals without cognitive concerns and normal neuropsychological performance (Montreal Cognitive Assessment [MoCA, HK version] ≥ 26). Final diagnoses for cognitively impaired participants were established by consensus among one neuroradiologist and two geriatricians, following established criteria: subjective cognitive decline according to Jessen [8] and MCI according to Petersen [9], and AD dementia according to McKhann [10].

AIBL_all cohort: Diagnostic classification was performed by a multidisciplinary panel (old-age psychiatrists, neurologist, geriatrician, neuropsychologists) using international consensus criteria: AD dementia by the National Institute of Neurological and Communicative Disorders and Related Disorders Association criteria [10], MCI by Winblad et al [11] and Petersen et al [9] criteria, subjective cognitive decline, CN, and other dementias. Consistent with the Winblad criteria [11], all MCI participants reported memory difficulties (self or informant). Baseline MCI diagnoses required scores ≥ 1.5 standard deviation below age-adjusted means on ≥ 1 neuropsychological test at reassessment. CN participants at baseline required ≥ 1.5 standard deviations below norms on ≥ 2 tests plus reported memory difficulties for reclassification to MCI at follow-up, a rigorous threshold reflecting MCI diagnostic instability and aligning with baseline protocols.

### MRI acquisition

#### Memory clinic

3-T MRI (Philips Achieva, 32-channel head coil; Philips Healthcare, Best, The Netherlands)) included three-dimensional (3D) T1-w magnetization prepared rapid gradient-echo sequence using repetition time (TR) = 6.8 ms, echo time (TE) = 3.2 ms, inversion time (TI) = 900 ms, slice thickness = 1.2 mm, flip angle = 8°, field of view (FOV) = 256 × 240 mm, matrix = 256 × 240; 3D T2-weighted FLAIR using TR = 4,800 ms, TE = 264 ms, TI = 1,650 ms, slice thickness = 1.12 mm, flip angle = 90°, FOV = 250 × 250 mm, matrix = 208 × 207. For PET acquisition, all subjects were required to fast for at least 6 h and rest in a dimmed room waiting for tracer injection. A bolus of ^18^F-Flutemetamol was administered intravenously (within 40 s) to the patients at a dosage of 185 MBq (approximately 5 mCi). The scanning started at 90 min after injection, using an integrated in-line PET/CT scanner (Discovery MI, GE Healthcare, Waukesha, Wisconsin, USA) with 3D list mode. Filtered back-projection reconstruction was used with a slice thickness of 2–4 mm, matrix size of 128 × 128, and a pixel size of 2 mm. A full-width half-maximum post-smoothing filter was applied, of not more than 5 mm. The duration of the scan lasted 30 min. Standardized uptake value ratio (SUVR) in 16 cortical regions was calculated with reference to the pons by automated software developed by the manufacturer (CortexID, GE Healthcare).

#### AIBL_all cohort

The imaging data included PET scanning with ^11^C-PiB/^18^F-AV45/^18^F-Flutemetamol and MRI. 3-T MRI from three centers (Trio, Skyra, and Verio, Siemens Healthineers, Erlangen, Germany) included a 3D magnetization-prepared rapid gradient-echo sequence (voxel size 1.2 × 1 × 1 mm^3^, TR/TE/TI = 2,300/2.98/900 ms, flip angle = 9°, FOV = 256 × 256 mm). Two 3D FLAIR protocols: (1) in-plane resolution = 0.98 × 0.98 mm, slice thickness = 0.9 mm, TR/TE/TI = 6,000/420/2100 ms, flip angle =  120°, FOV = 240 × 256 mm, and 176 slices; (2) in-plane after interpolation (factor of 2) = 0.5 × 0.5 mm, slice thickness = 1.0 mm, TR/ET/TI =  5,000/355/1,800 ms, flip angle = 120°, FOV = 256 × 256 mm, and 160 slices.

### MRI processing and WMH segmentation

MRI processing was performed on the United Imaging Artificial Intelligence (uAI) system. Automated WMH segmentation was conducted on 3D FLAIR images to quantify lesion volumes in four predefined regions: JVWMH region (JVWMH, ≤ 3 mm from the ventricular surface); periventricular WMH region (PVWMH, 3–13 mm from the ventricular surface); deep WMH region (DWMH, between the periventricular region and juxtacortical area); juxtacortical WMH region (JCWMH, ≤ 4 mm from the corticomedullary junction).

### Validation of WMH evaluation

To validate the accuracy of WMH assessment, Fazekas scores given by the uAI system were compared with the Fazekas scores independently rated by two radiologists. Discrepancies were resolved by consensus. Both raters were completely blinded to all clinical information, including diagnostic status, cognitive test scores, and Aβ-PET results. Periventricular and deep WMH were graded 0–3. Inter-rater intraclass correlation coefficients were 0.843 (95% confidence interval: 0.806–0.874) for PVWMH and 0.813 (95% confidence interval: 0.767–0.850) for DWMH.

Since JVWMH is defined by anatomical location (within 3 mm of the lateral ventricular surface) rather than by visual rating, traditional validation against visual scales is not applicable. To ensure the reliability of the automated segmentation, all automated WMH segmentations were visually inspected by a trained rater (blinded to clinical data) to confirm anatomical plausibility and exclude segmentation failures.

### PVS segmentation

PVS segmentation was performed on preprocessed T1w images using a Frangi filter-based algorithm. WM lesions were excluded from the PVS mask to avoid misclassification. The PVS volume was measured in the white matter (WM), basal ganglia (BG), hippocampus, and the sum of these structures (ALL). To account for interindividual brain size variability, PVS volume fraction (PVSVF; PVSVF = PVS volume/intracranial volume) was calculated and used for the following group comparison. During the process above, gray matter volume, including both deep gray matter structures and cerebellar gray matter, white matter volume (WMV), cerebrospinal fluid volume (CSFV), and intracranial volume were derived using the Computational Anatomy Toolbox 12 [12] (CAT12, http://www.neuro.uni-jena.de/cat/).

### PET processing and centiloid (CL) scaling

To enable cross-tracer comparability, all quantitative Aβ values were converted to CL units following the CL pipeline [13]. Specifically, T1w MRI and PET images of each participant were processed by Statistical Parametric Mapping 12 [14], including co-registration, quality control, segmentation, normalization, and extraction of Aβ-PET signals. After applying the global cortical target mask, SUVR values were computed from co-registered and normalized A-PET images by the REST toolbox [15] (http://www.restfmri.net/forum/REST_V1.8, version 1.8), with the whole cerebellum as reference. Linear equations converted ^18^F-AV45, ^18^F-Flutemetamol, and ^11^C-PiB SUVRs to CL units.

### Aβ positivity definition

In the AIBL_all cohort, as multiple tracers (¹¹C-PiB, ¹⁸F-Florbetapir, and ¹⁸F-Flutemetamol) were utilized, a CL scale to harmonize tracer-specific SUVR values was employed. CL > 12 corresponds to the transition from absence of pathology to early/subtle Aβ pathology. This threshold has been validated against post-mortem neuropathological examination [16], CSF Aβ42 abnormalities [17], and detection of early downstream pathophysiological changes in preclinical AD [18]. CL > 30 indicates the presence of established Aβ pathology with high certainty. This threshold aligns with moderate-to-frequent neuritic plaque burden at autopsy [19], optimal agreement with CSF p-tau/Aβ42 ratio and visual read positivity by expert rater [20]. Following the recent AMYPAD consortium recommendations [1], CL > 30 was applied to define Aβ positivity with high certainty in the AIBL_all cohort. The CL > 12 threshold was used to define Aβ positivity in sensitivity analyses or as a more exploratory cutoff to capture early amyloid accumulation.

In the memory clinic cohort, as only a single tracer (¹⁸F-Flutemetamol) was used, the validated tracer-specific SUVR threshold (≥ 0.62) was applied [21]. This approach is standard practice to avoid additional transformation and maintain the sensitivity/specificity established for that specific ligand.

### Statistical analysis

Analyses were conducted in SPSS (version 26; IBM Corp, Armonk, NY, USA). Prespecified outcomes were: (1) interaction effect between JVWMH and Aβ; (2) CI *versus* CN discrimination by JVWMH volume; (3) associations with cognition and longitudinal decline; and (4) mediation analyses of CSFV-cognition pathways.

Normality was assessed within each cohort using the Shapiro–Wilk test. Comparisons between groups employed independent-samples *t*-tests or Mann–Whitney *U*-tests for continuous variables, and χ² or Fisher exact tests for categorical variables. Multiple comparisons were controlled with Bonferroni correction within each analysis family. Continuous variables are presented as mean ± standard deviation or median (25th, 75th percentiles) depending on the normality of the data distribution; categorical variables are presented as frequency (percentage).

Two-way ANOVA was applied to analyze the interaction effect between JVWMH and Aβ, with continuous JVWMH data translating into high, medium, and low based on percentiles (specifically the 27th and 73rd percentiles). For diagnostic discrimination, primary logistic regression models included JVWMH volume as the predictor, adjusting for age, sex, and ICV. Secondary models incorporated other regional WMH volumes, Fazekas scores, and Aβ burden (expressed as SUVR or CL). The stepwise method was used for variable selection.

Hierarchical multiple regression evaluated predictors of cognitive performance (MoCA or MMSE). Block 1 included covariates (age, sex, and ICV); Block 2 included Aβ burden and/or CSFV; Block 3 included JVWMH, other regional WMH volumes, and Fazekas scores. For longitudinal analyses in the **AIBL_follow-up** cohort, the dependent variable was cognitive status according to a decrease in MMSE scores.

Partial correlations examined relationships among JVWMH volume, Aβ burden (SUVR/CL), and CSFV, controlling for age, sex, and ICV. Mediation analyses tested whether JVWMH volume mediated the effects of CSFV on cognition, adjusting for age, sex, and ICV. Indirect effects were estimated with bias-corrected bootstrapping (5,000 resamples).

**Fig. 2:**
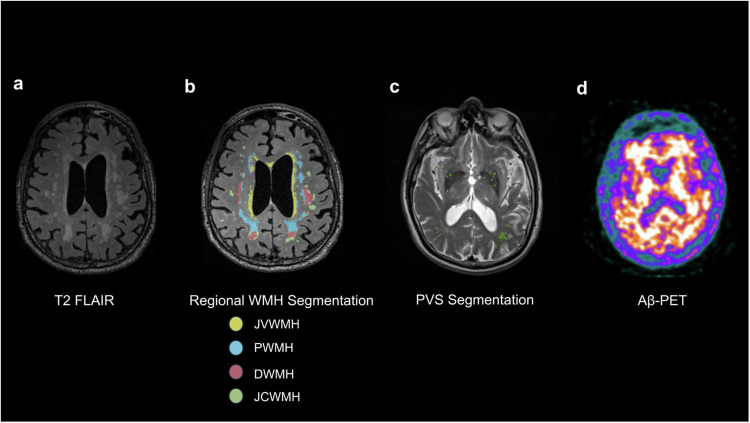
Multimodal imaging in a representative subject (**a**–**d**)

All analyses were conducted by researchers blinded to participant identifiers (including names, diagnoses, and clinical data) to ensure objectivity. The statistical significance was set at two-tailed α = 0.05. Multimodal imaging in a representative subject was shown in Fig. 2.

## Results

### Participant characteristics and group comparisons

Demographic data of all participants are summarized in Table 1. In both cohorts, there were no significant differences in sex, ICV, and volume of DWMH. The age, global Aβ burden, volume of JVWMH, volume of PVWMH, CSFV, Fazekas score of PVWMH, and Fazekas score of DWMH were significantly higher in the CI group compared to the CN group in both cohorts. CI participants had significantly lower MMSE/MoCA scores, and WMV compared to CN participants in both cohorts. In the AIBL_all cohort, the CI group exhibited a significantly higher prevalence of both CL > 12 and CI > 30. In the memory clinic cohort, the CI group showed a significantly higher percentage of Aβ positivity, and gray matter volume was significantly larger in the CN group compared to the CI group. However, no significant gray matter volume difference was observed between groups in the AIBL_all cohort.

**Table 1:** Demographics

	AIBL_all cohort				Memory clinic cohort			
	NC	CI	statistic	*p*	NC	CI	statistic	*p*
*N*	194	101			24	58		
Age (years)	73 (69.0, 76.0)	75 (71.0, 81.0)	-2.91	0.004^**^	65.92 ± 5.33	77.64 ± 8.18	-7.666	0.000^**^
Sex (no. of females, %)	117, 60.31%	51, 50.50%	2.609	0.106	9, 37.50%	34, 58.62%	3.036	0.081
MMSE	29 (28.0, 30.0)	26 (21.0, 28.0)	-10.018	0.000^**^	/	/	/	/
MoCA	/	/			28 (26.5, 28.5)	20 (12.0, 23.0)	-5.267	0.000^**^
Aβ burden (CL)	10.350 (-1.5, 26.7)	35.000 (13.9, 65.9)	-6.693	0.000^**^	/	/	/	/
CL > 12 (no., %)	91, 46.91%	78, 77.23%	24.955	0.000^**^	/	/	/	/
CL > 30 (no., %)	41, 21.13%	56, 55.45%	35.431	0.000^**^	/	/	/	/
Aβ burden (SUVR)	/	/			0.460 (0.4, 0.5)	0.645 (0.4, 0.8)	-2.835	0.005^**^
SUVR > 0.62 (no., %)	/	/			2, 8.33%	37, 63.79%	20.935	0.000^**^
GMV (cm^3^)	572.470 (541.9, 607.1)	558.600 (505.7, 617.4)	-1.584	0.113	579.29 ± 64.47	532.56 ± 49.64	3.545	0.001^**^
WMV (cm^3^)	464.370 (432.0, 492.9)	445.570 (404.0, 490.2)	-2.358	0.018^*^	464.55 ± 75.07	416.27 ± 53.13	3.302	0.001^**^
CSFV (cm^3^)	392.090 (345.1, 428.7)	442.250 (404.1, 489.6)	-5.853	0.000^**^	347.88 ± 78.85	400.00 ± 82.54	-2.635	0.010^*^
ICV (cm^3^)	1,415.590 (1,342.6, 1,521.9)	1,451.660 (1,358.1, 1,561.4)	-1.318	0.187	1,391.72 ± 191.00	1,348.82 ± 141.62	1.123	0.265
Volume of JVWMH (cm^3^)	2.168 (1.3, 3.8)	3.356 (1.9, 6.2)	-4.371	0.000^**^	3.030 (2.2, 4.4)	5.273 (4.0, 8.6)	-4.066	0.000^**^
Volume of periventricular WMH (cm^3^)	0.326 (0.1, 1.0)	0.923 (0.2, 3.9)	-3.317	0.001^**^	1.540 (0.9, 2.5)	3.226 (1.6, 8.5)	-3.006	0.003^**^
Volume of deep WMH (cm^3^)	0.312 (0.2, 0.7)	0.519 (0.2, 1.4)	-2.861	0.004^**^	0.089 (0.0, 0.4)	0.068 (0.0, 0.2)	-0.505	0.614
Volume of juxtacortical WMH (cm^3^)	0.051 (0.0, 0.2)	0.065 (0.0, 0.2)	-1.373	0.17	1.080 (0.4, 1.2)	0.711 (0.4, 1.3)	-0.927	0.354
Fazekas score of periventricular WMH	1 (1.0, 1.0)	1 (1.0, 2.0)	-3.304	0.001^**^	1 (1.0, 1.0)	1 (1.0, 2.0)	-3.304	0.001^**^
Fazekas score of deep WMH	1 (1.0, 1.0)	1 (1.0, 2.0)	-2.706	0.007^**^	1 (1.0, 1.0)	1 (1.0, 2.0)	-2.303	0.021^*^

Continuous variables are presented as mean ± SD or median (p25, p75) depending on the normality of the data distribution; categorical variables are presented as frequency (percentage)

^*^ *p* < 0.05, ^**^p < 0.01

### Interaction effect between JVWMH volume and Aβ status

No significant difference in JVWMH volume was observed between Aβ positive (Aβ+) and Aβ negative (Aβ-) individuals in either cohort (Supplementary Table S1). Two-way ANOVA analysis revealed no interaction effect between JVWMH and Aβ status, and this finding was consistent across both cohorts (AIBL_all cohort: *p* = 0.841; Memory clinic cohort: *p* = 0.074) (Supplementary Table S2). In the Supplementary Table S3, the two-way ANOVA within the Aβ+ subgroup was repeated as the sensitivity analysis, and the result remained consistent (AIBL_all cohort: *p* = 0.854).

### Associations of JVWMH volume with diagnostic status and cognitive performance

Logistic regression models, incorporating variables with significant intergroup differences (regional WMH volumes, WMH Fazekas scores, global Aβ burden), controlling for age, sex and ICV, revealed significant positive associations between JVWMH volume and diagnostic status (CN *versus* CI) in both AIBL_all (β = 0.202, *p* < 0.01; pseudo *R*² = 0.286) and memory clinic cohorts (β = 0.674, *p* = 0.005; pseudo *R*² = 0.749) (Table 2).

**Table 2:** Associations of JVWMH volume and diagnostic status using a logistic regression model

	Pseudo *R*^2^	β	SE	*p*
AIBL_all cohort logistic regression model				
Aβ burden (CL)	0.286	0.028	0.005	0.000^**^
Volume of JVWMH		0.202	0.05	0.000^**^
Memory clinic cohort logistic regression model				
Age	0.749	0.247	0.072	0.001^**^
Aβ burden (SUVR)		12.364	3.901	0.002^**^
Volume of JVWMH		0.674	0.24	0.005^**^

Dependent variable: CN or CI

Pseudo−*R*² (Nagelkerke *R*²) is reported for logistic regression models

Stepwise selection was used to retain significant predictors in the final models

^**^p < 0.01

Hierarchical multiple regression modeling further demonstrated that greater JVWMH volume significantly predicted poorer cognitive performance in the AIBL_all cohort (β = -0.392, *p* = 0.002; adjusted R² = 0.246), but not in the memory clinic cohort (β = 0.304, *p* = 0.146; adjusted *R*² = 0.484; Table 3).

**Table 3:** Associations of JVWMH volume and cognitive performance using a hierarchical multiple regression model

	Adj.*R*^2^	β	SE	statistic	*p*
AIBL_all cohort hierarchical multiple regression					
Age	0.246	-0.11	0.039	-1.799	0.073
Sex		-0.051	0.622	-0.668	0.505
ICV		0.224	0.002	2.745	0.006^**^
Aβ burden (CL)		-0.264	0.006	-4.759	0.000^**^
Volume of JVWMH		-0.392	0.148	-3.187	0.002^**^
Volume of PVWMH		0.176	0.105	1.221	0.223
Volume of DWMH		0.207	0.427	2.016	0.045*
Volume of JCWMH		-0.223	0.181	-1.83	0.068
Fazekas score of PVWMH		-0.125	0.843	-1.023	0.307
Fazekas score of DWMH		0.149	0.881	1.212	0.227
Memory clinic cohort hierarchical multiple regression					
Age	0.484	0.515	0.005	5.146	0.000^**^
Sex		-0.103	0.099	-0.948	0.346
ICV		0.06	0	0.503	0.616
Aβ burden (SUVR)		0.358	0.001	4.047	0.000^**^
Volume of JVWMH		0.304	0.024	1.47	0.146
Volume of PVWMH		-0.297	0.015	-1.342	0.184
Volume of DWMH		-0.198	0.211	-1.213	0.229
Volume of JCWMH		0.142	0.056	1.101	0.275
Fazekas score of PVWMH		0.27	0.107	2.059	0.043^*^
Fazekas score of DWMH		-0.132	0.123	-0.894	0.375

Dependent variable: MMSE/MoCA scores

The data presented in the table is based on the Block 3 model

^*^ *p* < 0.05, ^**^ *p* < 0.01

Longitudinal analysis in the AIBL_follow-up cohort (Table 4) indicated that baseline JVWMH volume significantly predicted cognitive decline over time (β = -0.773, *p* = 0.005; adjusted *R*² = 0.319). The differences in follow-up time and MMSE scores at baseline between groups were not statistically significant (Supplementary Table S5). During follow-up, 7 out of 84 subjects experienced cognitive decline, defined as a reduction of ≥ 3 points on the MMSE. Among these 7 individuals, 2 were classified as CN, 1 as MCI, and 4 as AD at baseline. Supplementary Fig. S1 reveals a significant increase in JVWMH volume over time in the full cohort. In contrast, although the decline subgroup (*n* = 4) showed a similar upward trend, the result was not statistically significant, a finding that may be attributable to the limited sample size and low statistical power.

**Table 4:** Associations of JVWMH volume and cognitive decline using a hierarchical multiple regression model

	Adj.*R*^2^	β	SE	statistic	*p*
AIBL _follow−up cohort hierarchical multiple regression					
Age	0.319	0.146	0.006	1.339	0.185
Sex		-0.282	0.077	-2.054	0.044^*^
ICV		0.07	0	0.473	0.638
Follow−up time		0.252	0.003	2.718	0.008^**^
MMSE at baseline		-0.582	0.01	-4.592	0.000^**^
Aβ burden (CL)		0.096	0.001	0.949	0.346
Volume of JVWMHs		-0.773	0.023	-2.912	0.005^**^
Volume of periventricular WMHs		-0.539	0.014	-2.462	0.016^*^
Volume of deep WMHs		-0.472	0.098	-2.069	0.042^*^
Volume of juxtacortical WMHs		0.618	0.058	2.007	0.049^*^
Fazekas score of periventricular WMHs		0.101	0.089	0.585	0.56
Fazekas score of deep WMHs		0.827	0.123	4.081	0.000^**^

Dependent variable: cognitive status according to a decrease in MMSE scores

The data presented in the table is based on the Block 3 model

^*^ *p* < 0.05, ^**^ *p* < 0.01

### Perivascular space metrics and mediation effects of JVWMH

**Fig. 3:**
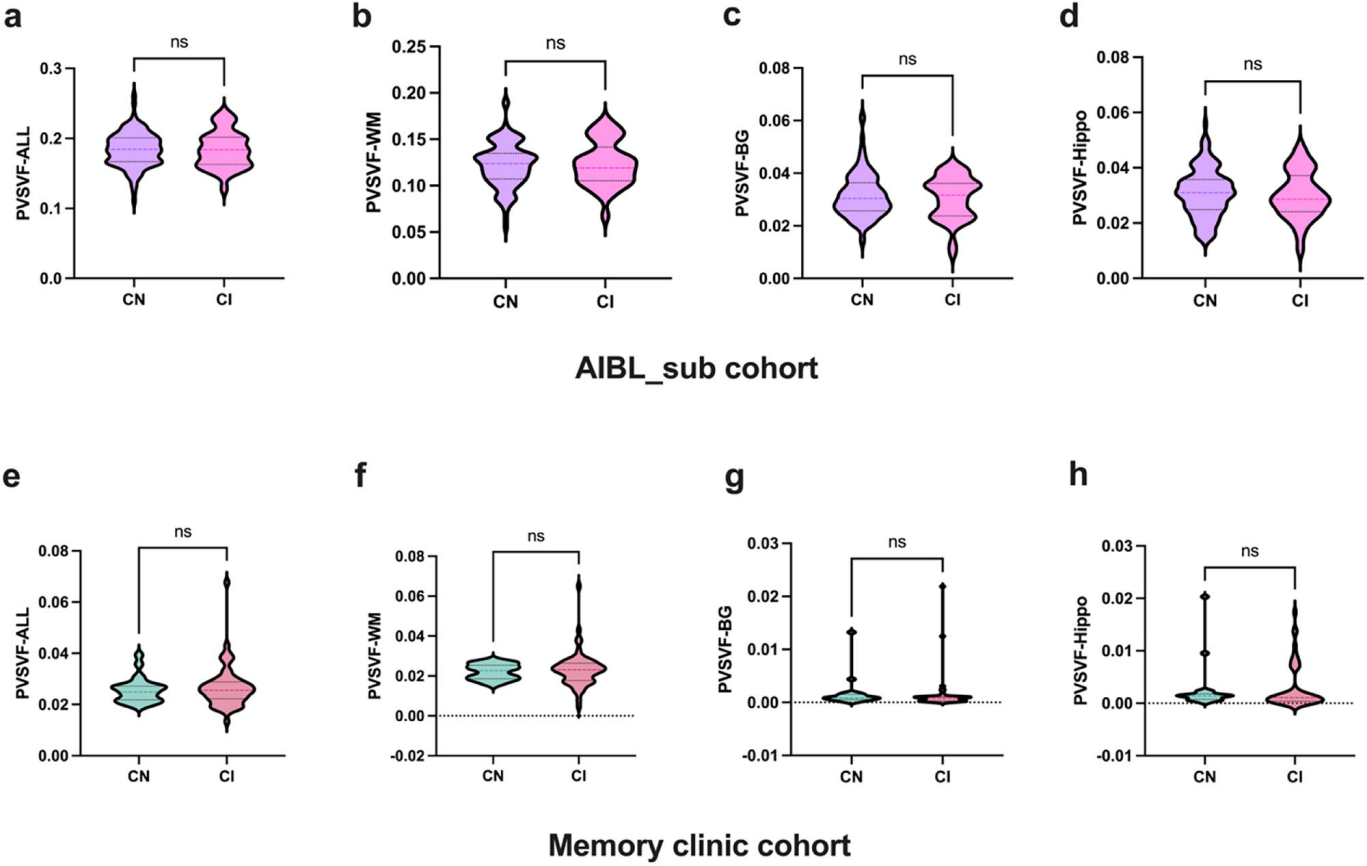
Perivascular space metrics comparisons between CN and CI groups in the AIBL_sub cohort (**a**–**d**) and in the memory clinic cohort (**e**–**h**)

Comparisons of PVSVF across multiple regions (PVSVF_ALL, PVSVF_WM, PVSVF_BG, PVSVF_Hippo) between CN and CI groups in both AIBL_sub and memory clinic cohorts revealed no significant differences (Fig. 3).

**Fig. 4:**
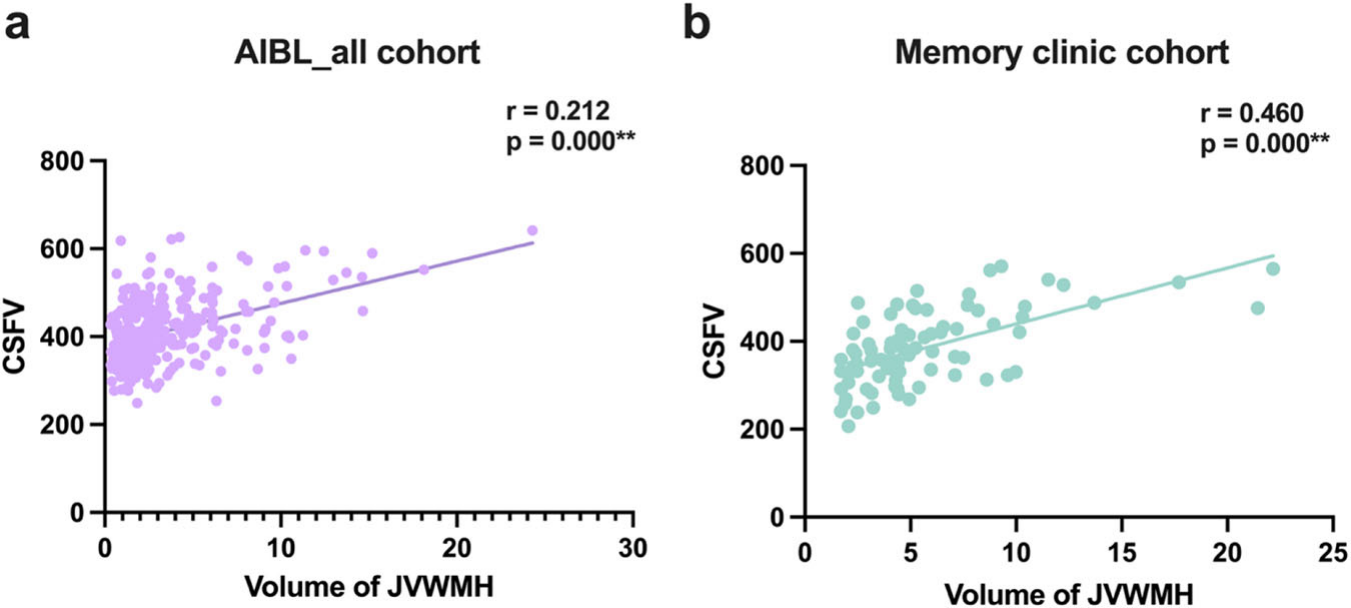
Correlations between CSFV and the volume of JVWMH (**a**, **b**)

Hierarchical regression models (Table 5) identified a consistent positive association between JVWMH volume and CSFV across cohorts, with no significant relationship observed between JVWMH and global Aβ burden. Partial correlation analyses in Fig. 4 corroborated this specific JVWMH-CSFV association (AIBL_all cohort: *r* = 0.212, *p* < 0.01; memory clinic cohort: *r* = 0.460, *p* < 0.01).

**Table 5:** Associations of JVWMH volume and CSFV using a hierarchical multiple regression model

	Adj.*R*^2^	β	SE	statistic	*p*
AIBL_all cohort hierarchical multiple regression					
Age	0.285	0.303	0.03	5.229	0.000^**^
Sex		-0.254	0.453	-3.652	0.000^**^
ICV		0.226	0.002	2.753	0.006^**^
Aβ burden (CL)		0.064	0.005	1.247	0.213
CSFV		0.263	0.004	3.305	0.001^**^
Memory clinic cohort hierarchical multiple regression					
Age	0.421	0.288	0.044	2.845	0.006^**^
Sex		0.015	0.894	0.131	0.896
ICV		0.024	0.004	0.15	0.881
Aβ burden (SUVR)		0.113	2.053	1.304	0.196
CSFV		0.525	0.006	3.879	0.000^**^

Dependent variable: volume of JVWMH

The data presented in the table is based on the Block 3 model

^**^ *p* < 0.01

**Fig. 5:**
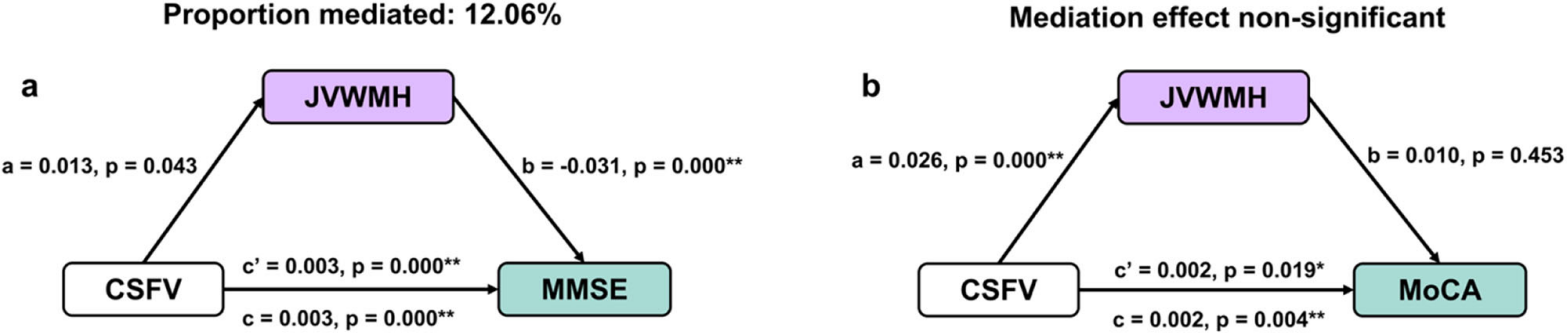
Mediation effects of JVWMH in the CSFV-cognition pathway (**a**, **b**)

Mediation analyses demonstrated that JVWMH significantly mediated the relationship between CSFV and cognition in the AIBL_all cohort (proportion mediated: 12.06%; total effect: *c* = 0.00, *p* < 0.01; direct effect: *c*' = 0.003, *p* < 0.01; indirect effect: *a* × *b* = 0.058). No significant mediation effect was detected in the memory clinic cohort for the CSFV-cognition pathway (Fig. 5).

The summary of key statistical findings was shown in the Supplementary Table S6.

## Discussion

Across two independent cohorts, JVWMH offered diagnostic and prognostic value and was correlated with CSFV. These findings support JVWMH as a practical, spatially specific marker of white matter pathology, though the underlying mechanisms remain uncertain.

Conventional PVWMH are likely lesions of non-vascular origin, distinct from subcortical and deep white matter abnormalities [22–24]. Growing evidence suggests that some WMHs may arise partly from neurodegenerative processes, not solely vascular disease [25, 26]. Periventricular regions exhibit particular vulnerability to hemodynamic fluctuations in distal arterial territories. Ventricular geometry may influence signal abnormality distribution along ventricular margins, with mechanical forces potentially compromising ependymal integrity and facilitating dysregulated fluid exchange [27]. Thus, JVWMH may result from CSF pressure-driven white matter edema or impaired subependymal interstitial fluid reabsorption [23]. In CI patients, JVWMH expansion promotes CSF seepage into periventricular tissue. Our study confirmed that CI individuals exhibited significantly larger CSFV (*i.e.*, increased CSFV, often reflecting ventricular enlargement or parenchymal atrophy) and greater JVWMH burden than CN controls, consistent with prior reports [28–30]. However, the mean JVWMH volume we observed in CI groups was lower than that previously reported in AD patients [31]. This discrepancy likely reflects inclusion of MCI patients, as JVWMH volume increased stepwise from CN to MCI to AD (Supplementary Fig. S2), with CI group means falling intermediate between MCI and AD. Differences in segmentation approaches and FLAIR parameters may also contribute, as longer TR/TI improves CSF suppression and lesion-to-white matter contrast, potentially yielding higher absolute volumes [32, 33].

The glymphatic system, the brain’s primary metabolic waste clearance pathway, relies on arterial pulsation to drive CSF influx along PVS, flowing parallel to blood vessels [3]. PVS dilation, a key indicator of glymphatic function, is consistently associated with greater WMH severity [34, 35]. Despite significant CSFV differences, neither cohort showed significant PVS dilation differences between CI and CN groups. While some studies report increased PVS dilation in AD *versus* controls [4–6], this finding may not extend to comparisons involving MCI. PVS dilation may primarily occur in the late stages of AD, whereas the CI group in this study included a large proportion of MCI individuals. In these cases, glymphatic dysfunction may manifest preferentially as increased CSFV rather than macroscopic PVS alterations. Of note, while the association between JVWMH and CSFV is consistent with processes occurring at the CSF-parenchyma interface, such as ependymal disruption or impaired CSF exchange, it does not constitute evidence of glymphatic dysfunction. Alternative or concomitant explanations may include microvascular ischemia, ventricular geometry-related stress, age-related tissue fragility, and atrophy.

Logistic regression demonstrated that JVWMH volume significantly discriminated CI from CN groups, whereas non-JVWMH volumes and Fazekas scores lacked significant discriminatory power. Hierarchical regression revealed significant JVWMH-cognition associations in the AIBL_all cohort, and longitudinal analyses confirmed JVWMH's predictive value for cognitive decline. All models included global Aβ burden; although Aβ pathology significantly impacted cognition, JVWMH retained independent diagnostic utility. Most previous WMH topography studies employed data-driven methods, consistently identifying patterns separating deep from periventricular WMHs and posterior from anterior WMHs [30, 36]. These studies revealed that WMHs exhibit regional associations with cognitive symptoms. For instance, periventricular WMHs generally exhibit stronger correlations with cognitive dysfunction than deep WMHs [30, 36, 37]. Posterior periventricular WMHs are notably elevated in AD patients *versus* cognitively unimpaired individuals [38]. Critically, co-occurring WMH and Aβ pathology accelerated cognitive decline in healthy controls [39, 40]. Furthermore, anterior subcortical WMH pattern exerts cross-sectional and longitudinal effects on all-cause dementia risk [41]. In Yang et al's study, JVWMH significantly predicted impairment in categorical verbal fluency and forward digit span tasks. Similarly, PVWMH volume was linked to reduced performance on categorical verbal fluency and word list memory tests. Instead, DWMH volume demonstrated no associations with any cognitive measures.

We further investigated the mediating role of JVWMH in the relationships between CSFV and cognitive performance. Partial correlation analyses revealed significant associations between CSFV and JVWMH burden. Across both cohorts, JVWMH volume demonstrated strong positive correlations with CSFV. In the AIBL_all cohort, JVWMH exhibited partial mediation effects in CSFV-cognition pathways. The mediation effect was not significant in the Memory clinic cohort. This inconsistency may reflect cohort heterogeneity, such as different metrics (SUVR *versus* CL, MoCA *versus* MMSE), differential MCI/AD ratios, vascular comorbidity profiles, and limited ethnic diversity. Reliance on global cognitive screening rather than domain-specific assessments may have obscured nuanced relationships. MoCA provides more extensive executive function assessment—domains preferentially affected by periventricular damage [42, 43], whereas MMSE emphasizes orientation and memory. Given the established association between periventricular white matter damage and frontal-subcortical circuit dysfunction [44], JVWMH may disrupt the integrity of these pathways, leading to poorer performance on tasks requiring cognitive flexibility and retrieval strategies. Additionally, the maintenance and manipulation of attention (as assessed by the forward digit span tasks) also rely on the integrity of frontal-subcortical and parietal networks [45]. The impact of JVWMH on the subependymal region may interfere with neural networks related to attentional processes [31]. Future studies should employ comprehensive neuropsychological batteries that assess executive function and attention to better characterize the specific cognitive profile associated with JVWMH.

While prior studies examining WMH, Aβ, and CI interrelationships remain inconclusive, Ottoy et al reported that vascular burden influences cognition primarily through localized neurodegeneration rather than Aβ pathology [46]. Hong et al postulated that cerebral small vessel disease affects cognition via glymphatic dysfunction, independent of Aβ along the AD continuum [4]. Extending these findings, our study provides novel evidence that JVWMH exerts an independent predictive effect on cognition unmodulated by Aβ pathology, suggesting these pathologies contribute to cognitive decline through distinct pathways. The topographical specificity of JVWMH reflects CSF seepage into periventricular parenchyma, a mechanism closely linked to glymphatic dysfunction, aligning with existing literature [4, 47, 48].

The spatial hierarchical framework of WMHs challenges the conventional framework and has highlighted its theoretical and clinical values in the current study. It proposes a new pathological theory, CSF dynamics-dominant WMH pathogenesis, and addresses the association between JVWMH and CSF extravasation. The diagnostic and predictive value of JVWMH in cognitive decline and as a mediator linking CSFV to CI suggests the potential role of JVWMH as an early diagnostic biomarker. Thus, early intervention of WMHs during midlife represents a critical neuroprotective approach, whereby management of education [49] and vascular risks [50] may decelerate WMHs pathogenesis, ultimately limiting downstream neurodegeneration and AD pathological exacerbation [51].

There are several limitations to this study. First, we lacked direct glymphatic measures. Second, mediation analyses were exploratory and cohort-specific. Third, 3-T MRI resolution limits PVS quantification. Fourth, the memory clinic cohort was smaller, which may limit power and generalizability. Fifth, cross-cohort differences in MRI acquisition protocols may introduce potential bias in automated WMH segmentation. Specifically, variations in TR/TE can affect signal-to-noise ratio and lesion conspicuity, while suboptimal TR/TI combinations (*e.g*., below 9,000/2,400 ms) may reduce lesion-to-white matter contrast in FLAIR imaging, potentially influencing measured WMH volumes [9, 10], including absolute JVWMH volumes as shown in Supplementary Table 6. To minimize these effects, all primary analyses were conducted independently within each cohort, avoiding direct pooling of raw volumes, and a unified segmentation pipeline was applied across all images. Sensitivity analyses within the AIBL_all cohort confirmed that the JVWMH-cognition association did not differ significantly between protocols (*p* = 0.483; Supplementary Table S4), supporting the robustness of our findings. Nonetheless, residual confounding from protocol heterogeneity cannot be entirely excluded. Sixth, the use of different cognitive screening instruments (MMSE in AIBL and MoCA in the memory clinic) and amyloid quantification methods (CL and SUVR) between cohorts may affect the results. This methodological discrepancy may have contributed to the observed differences in effect sizes and statistical significance, as MoCA, showing greater sensitivity to executive function, may better capture JVWMH-related deficits compared to MMSE. Future studies should consider harmonized acquisition protocols or advanced harmonization techniques (*e.g*., ComBat) to mitigate such biases, pair JVWMH with direct CSF/glymphatic indices (*e.g.*, diffusion “analysis along the perivascular space”), higher-field PVS morphometry, and vascular risk adjustment.

In conclusion, JVWMH provides diagnostic and prognostic information beyond conventional WMH measures in AD. The JVWMH-CSFV correlation is consistent with CSF-parenchyma interface processes but does not validate JVWMH as a glymphatic marker or establish a mechanism. These findings support reporting spatially stratified WMH alongside conventional measures in AD studies.

## Supplementary information

**Additional File 1: Table S1.** Comparisons of regional WMH volume according to Aβ status. **Table S2.** Two-way ANOVA testing the interaction effect between JVWMH and Aβ status. **Table S3.** Two-way ANOVA testing interaction between JVWMH and Aβ burden in the Aβ+ population. **Table S4.** Two-way ANOVA testing interaction between JVWMH and different FLAIR protocols. **Table S5.** Demographics in the AIBL_follow-up cohort. **Table S6:** Summary of Key Statistical Findings. **Fig. S1.** The change of JVWMH volume over time. **Fig. S2.** A graded increase in JVWMH volume from CN to MCI to AD.

## Data Availability

The datasets used and analyzed during the current study are available from one of the authors (HKFM) on reasonable request after evaluation by the authors and, if applicable, by the local ethics authority.

## Abbreviations

3D: Three-dimensional
Aβ: β-amyloid
AD: Alzheimer disease
AIBL: Australian Imaging, Biomarkers and Lifestyle
CI: Cognitive impairment
CL: Centiloid
CN: Cognitively normal
CSF: Cerebrospinal fluid
CSFV: Cerebrospinal fluid volume
DWMH: Deep white matter hyperintensities
FLAIR: Fluid-attenuated inversion recovery
FOV: Field of view
JCWMH: Juxtacortical white matter hyperintensities
JVWMH: Juxtaventricular white matter hyperintensities
MCI: Mild cognitive impairment
MRI: Magnetic resonance imaging
PET: Positron emission tomography
PVS: Perivascular space
PVSVF: Perivascular space volume fraction
PVWMH: Periventricular white matter hyperintensities
SUVR: Standardized uptake value ratios
TE: Echo time
TI: Inversion time
TR: Repetition time
WM: White matter
WMHs: White matter hyperintensities
WMV: White matter volume
