# Supplementary material for: Topography of Juxtaventricular white matter hyperintensities and cognitive associations in Alzheimer's disease: a dual-cohort study
Source: Eur Radiol Exp. 2026 Jul 29;10:109. doi: 10.1186/s41747-026-00776-1 (PMC13421530; doi:10.1186/s41747-026-00776-1)
Supplement: Supplementary file 1 — Additional File 1: Table S1. Comparisons of regional WMH volume according to Aβ status. Table S2. Two-way ANOVA testing the interaction effect between JVWMH and Aβ status. Table S3. Two-way ANOVA testing interaction between JVWMH and Aβ burden in the Aβ+ population. Table S4. Two-way ANOVA testing interaction between JVWMH and different FLAIR protocols. Table S5. Demographics in the AIBL_follow-up cohort. Table S6: Summary of Key Statistical Findings. Fig. S1. The change of JVWMH volume over time. Fig. S2. A graded increase in JVWMH volume from CN to MCI to AD. [file 41747_2026_776_MOESM1_ESM.pdf]

# Topography of Juxtaventricular White Matter Hyperintensities and Cognitive Associations in Alzheimer Disease: A Dual-Cohort Study

## Electronic Supplementary material

**Table S1** Comparisons of regional WMH volume according to Aβ status

| | AIBL_all cohort | | statistic | p | Memory clinic cohort | | statistic | p |
|---|---|---|---|---|---|---|---|---|
| | Aβ- | Aβ+ | | | Aβ- | Aβ+ | | |
| N | 126 | 169 | | | 43 | 39 | | |
| Volume of juxtaventricular WMH ($cm^3$) | 2.407(1.3, 4.5) | 2.513(1.5, 4.8) | -0.686 | 0.493 | 4.365(2.5, 6.0) | 5.109(3.9, 7.8) | -1.685 | 0.092 |
| Volume of periventricular WMH ($cm^3$) | 0.363(0.1, 1.9) | 0.498(0.1, 2.2) | -0.316 | 0.752 | 2.271(1.2, 4.3) | 2.449(1.4, 9.6) | -1.305 | 0.192 |
| Volume of deep WMH ($cm^3$) | 0.056(0.0, 0.3) | 0.052(0.0, 0.1) | -0.623 | 0.533 | 0.040(0.0, 0.2) | 0.115(0.0, 0.4) | -1.64 | 0.101 |
| Volume of juxtacortical WMH ($cm^3$) | 0.380(0.2, 1.0) | 0.399(0.2, 0.9) | -0.072 | 0.942 | 0.927(0.4, 1.2) | 0.788(0.4, 1.3) | -0.311 | 0.756 |

* p<0.05, ** p<0.01

Continuous variables are presented as mean ± SD or median (p25, p75) depending on the normality of data distribution; categorical variables are presented as frequency (percentage)

**Table S2** Two-way ANOVA testing interaction effect between JVWMH and Aβ status

| | | **Sum of squares** | **df** | **Mean square** | **F** | **p** |
|---|---|---|---|---|---|---|
| **AIBL_all cohort** | Intercept | 139.857 | 1 | 139.857 | 7.903 | 0.006 ** |
| | Volume of juxtaventricular WMH (cm3) | 183.723 | 2 | 91.862 | 5.191 | 0.007 ** |
| | CL | 248.214 | 2 | 124.107 | 7.013 | 0.001 ** |
| | Volume of juxtaventricular WMH (cm3) * CL | 25.065 | 4 | 6.266 | 0.354 | **0.841** |
| | Sex | 88.081 | 1 | 88.081 | 4.977 | 0.027 * |
| | Age | 57.04 | 1 | 57.04 | 3.223 | 0.075 |
| | ICV | 213.021 | 1 | 213.021 | 12.037 | 0.001 ** |
| | Residual | 2760.682 | 156 | 17.697 | | |
| **Memory clinic cohort** | Intercept | 0.283 | 1 | 0.283 | 2.511 | 0.118 |
| | Volume of juxtaventricular WMH (cm3) | 0.264 | 2 | 0.132 | 1.168 | 0.317 |
| | SUVR | 1.202 | 2 | 0.601 | 5.327 | 0.007 ** |
| | Volume of juxtaventricular WMH (cm3) * SUVR | 1.008 | 4 | 0.252 | 2.233 | **0.074** |
| | Age | 2.208 | 1 | 2.208 | 19.57 | 0.000 ** |
| | Sex | 0.114 | 1 | 0.114 | 1.007 | 0.319 |
| | ICV | 0.035 | 1 | 0.035 | 0.306 | 0.582 |
| | Residual | 7.898 | 70 | 0.113 | | |

* p<0.05, ** p<0.01

Controlling for age, sex and ICV

Continuous JVWMH data translated into categorical groups (high, medium, low)

**Table S3** Two-way ANOVA testing interaction between JVWMH and Aβ burden in Aβ+ population

| | | Sum of squares | df | Mean square | F | p |
|---|---|---|---|---|---|---|
| **AIBL_all cohort** | Intercept | 0.087 | 1 | 0.087 | 1.398 | 0.25 |
| | Volume of juxtaventricular WMH (cm3) | 0.052 | 2 | 0.026 | 0.414 | 0.666 |
| | CL | 0.108 | 2 | 0.054 | 0.865 | 0.436 |
| | Volume of juxtaventricular WMH (cm3) * CL | 0.083 | 4 | 0.021 | 0.332 | **0.854** |
| | Sex | 0.05 | 1 | 0.05 | 0.806 | 0.379 |
| | Age | 0.005 | 1 | 0.005 | 0.08 | 0.78 |
| | ICV | 0.052 | 1 | 0.052 | 0.826 | 0.374 |
| | Residual | 1.31 | 21 | 0.062 | | |

* $p<0.05$, ** $p<0.01$

Controlling for age, sex and ICV

Continuous JVWMH data translated into categorical groups (high, medium, low)

**Table S4** Two-way ANOVA testing interaction between JVWMH and different FLAIR protocols

| | | Sum of squares | df | Mean square | F | p |
|---|---|---|---|---|---|---|
| **AIBL_all cohort** | Intercept | 0.037 | 1 | 0.037 | 0.18 | 0.672 |
| | Volume of juxtaventricular WMH (cm3) | 1.791 | 2 | 0.896 | 4.338 | 0.014* |
| | FLAIR type | 0.229 | 1 | 0.229 | 1.109 | 0.293 |
| | Volume of juxtaventricular WMH (cm3) * FLAIR type | 0.301 | 2 | 0.151 | 0.729 | **0.483** |

| | | | | | | |
|---|---|---|---|---|---|---|
| | Sex | 0.631 | 1 | 0.631 | 3.057 | 0.081 |
| | Age | 0.254 | 1 | 0.254 | 1.23 | 0.268 |
| | ICV | 0.14 | 1 | 0.14 | 0.676 | 0.412 |
| | Residual | 58.841 | 285 | 0.206 | | |

* p<0.05, ** p<0.01

Controlling for age, sex and ICV

Continuous JVWMH data translated into categorical groups (high, medium, low)

**Table S5** Demographics in the AIBL_follow-up cohort

| | **AIBL_follow-up** | | | |
|---|---|---|---|---|
| | stable | decline | statistic | p |
| N | 77 | 7 | | |
| Diagnosis as CN at baseline (no., %) | 61, 79.22% | 2, 28.57% | / | / |
| Diagnosis as MCI at baseline (no., %) | 11, 14.29% | 1, 14.29% | / | / |
| Diagnosis as AD at baseline (no., %) | 5, 6.49% | 4, 57.14% | / | / |
| Age (years) | 73 (69.0, 76.0) | 73 (70.0, 81.0) | -0.495 | 0.621 |
| Sex (no. of females, %) | 42, 54.55% | 7, 100.00% | 5.455 | 0.020* |
| MMSE at baseline | 29 (28.0, 30.0) | 28 (21.0, 30.0) | -0.97 | 0.332 |
| MMSE at follow-up | 29 (28.0, 30.0) | 23 (16.0, 26.0) | -4.024 | 0.000** |
| follow-up time | 18 (18.0, 18.0) | 18 (18.0, 18.0) | -1.611 | 0.107 |
| Aβ burden (CL) | 11.220 (-1.2,32.5) | 38.940 (6.9,68.5) | -1.319 | 0.187 |
| CL > 12 (no., %) | 38, 49.35% | 5, 71.43% | 1.252 | 0.263 |
| CL > 30 (no., %) | 20, 25.97% | 4, 57.14% | 3.055 | 0.081 |
| GMV ($cm^3$) | 587.43±58.25 | 544.58±68.08 | 1.838 | 0.07 |
| WMV ($cm^3$) | 476.415 (447.9, 518.0) | 438.050 (399.1, 456.9) | -2.524 | 0.012* |
| CSFV ($cm^3$) | 398.32±74.90 | 372.02±64.36 | 0.898 | 0.372 |
| ICV ($cm^3$) | 1442.420 (1352.2, 1605.9) | 1359.080 (1250.1, 1444.8) | -1.983 | 0.047* |
| Volume of juxtaventricular WMH ($cm^3$) | 2.225 (1.3, 4.3) | 6.139 (1.3, 6.3) | -1.076 | 0.282 |
| Volume of periventricular WMH ($cm^3$) | 0.355 (0.1, 1.7) | 1.690 (0.2, 5.5) | -1.27 | 0.204 |
| Volume of deep WMH ($cm^3$) | 0.051 (0.0, 0.2) | 0.126 (0.0, 0.2) | -0.397 | 0.692 |
| Volume of juxtacortical WMH ($cm^3$) | 0.341 (0.2, 1.1) | 1.331 (0.3, 2.0) | -1.27 | 0.204 |
| Fazekas score of periventricular WMH | 1 (1.0, 1.5) | 2 (1.0, 2.0) | -1.753 | 0.08 |
| Fazekas score of deep WMH | 1 (1.0, 1.0) | 1 (1.0, 2.0) | -1.59 | 0.112 |

* p<0.05, ** p<0.01

Continuous variables are presented as mean ± SD or median (p25, p75) depending on the normality of data distribution; categorical variables are presented as frequency (percentage).

**Table S6** Summary of Key Statistical Findings

| Main Analysis type | Key findings | Statisitically evidence |
|---|---|---|
| Two-way ANOVA | The effect of JVWMH volume on cognition was not significantly vary by Aβ status | AIBL_all cohort: $p = 0.841$; Memory clinic cohort: $p = 0.074$ |
| Logistic regression model | JVWMH volume independently associated with cognitive status (e.g., CN/CI) cross-sectionally | AIBL_all cohort: $\beta = 0.202$, $p < 0.01$; pseudo $R^2 = 0.286$; memory clinic cohort: $\beta = 0.674$, $p = 0.005$; pseudo $R^2 = 0.749$ |
| Hierarchical Multiple Regression | (1) JVWMH volume independently predicted cognitive performance (e.g., MMSE/MoCA) cross-sectionally and longitudinally; (2) JVWMH volume independently associated with CSFV | (1) AIBL_all cohort: $\beta = -0.392$, $p = 0.002$, adjusted $R^2 = 0.246$; memory clinic cohort: $\beta = 0.304$, $p = 0.146$, adjusted $R^2 = 0.484$; (2) AIBL_follow-up cohort: $\beta = -0.773$, $p = 0.005$, adjusted $R^2 = 0.319$ |
| Partial correlation | CSFV and signifcantly associated with JVWMH volume | AIBL_all cohort: $r = 0.212$, $p < 0.01$; memory clinic cohort: $r = 0.460$, $p < 0.01$ |
| Mediation analysis | JVWMH exhibited partial mediation effects in CSFV-cognition pathways | proportion mediated: 12.06%; total effect: $c = 0.00$, $p < 0.01$; direct effect: $c' = 0.003$, $p < 0.01$; indirect effect: $a*b = 0.058$ in the AIBL_all cohort; No significant mediation effect was detected in the memory clinic cohort |

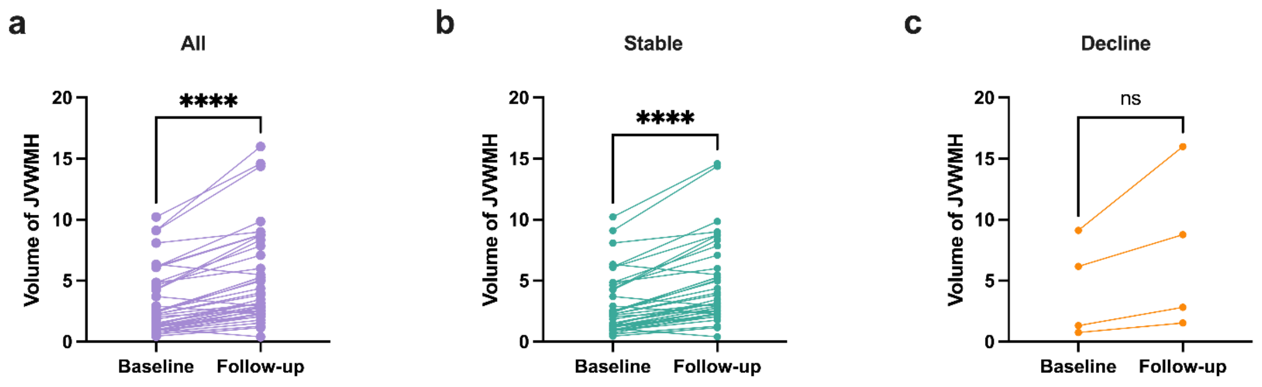


**Fig. S1** The change of JVWMH volume overtime

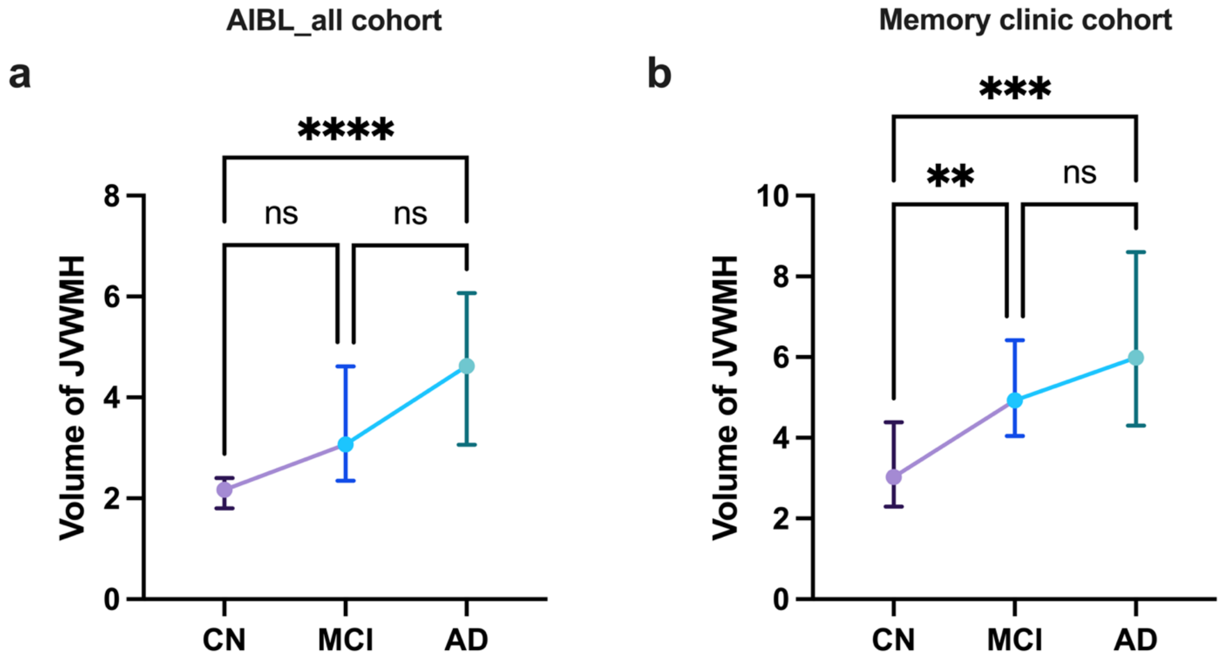


**Fig. S2** A graded increase in JVWMH volume from CN to MCI to AD
